# Biphasic Dynamics of Inflammatory Markers Following Hemodialysis Initiation: Results From the International MONitoring Dialysis Outcome Initiative

**DOI:** 10.1016/j.ekir.2022.10.020

**Published:** 2022-11-16

**Authors:** Dalia E. Yousif, Xiaoling Ye, Stefano Stuard, Juan Berbessi, Adrian M. Guinsburg, Len A. Usvyat, Jochen G. Raimann, Jeroen P. Kooman, Frank M. van der Sande, Neill Duncan, Kevin J. Woollard, Rupert Bright, Charles Pusey, Vineet Gupta, Joachim H. Ix, Peter Kotanko, Rakesh Malhotra

**Affiliations:** 1 Division of Nephrology, Department of Medicine, Soba University Hospital, Khartoum, Sudan; 2 Research Division, Renal Research Institute, New York, New York, USA; 3 Fresenius Medical Care EMEA, Bad Homburg, Germany; 4 Fresenius Medical Care Global Medical Office, Buenos Aires, Argentina; 5 Fresenius Medical Care, Waltham, Massachusetts, USA; 6 Division of Nephrology, Department of Internal Medicine, Maastricht University Medical Center, Maastricht, the Netherlands; 7 Faculty of Medicine, Imperial College London, London, UK; 8 Division of Hospital Medicine, Department of Medicine, University of California San Diego, San Diego, California, USA; 9 Herbert Wertheim School of Public Health, University of California San Diego, San Diego, California, USA; 10 Nephrology Section, Veteran Affairs San Diego Healthcare System, La Jolla, California, USA; 11 Division of Nephrology and Hypertension, University of California San Diego, San Diego, California, USA; 12 Division of Nephrology, Department of Medicine, Icahn School of Medicine at Mount Sinai, New York, New York, USA

**Keywords:** albumin, dynamics, end-stage kidney disease, inflammation, mortality, neutrophil-lymphocyte ratio

## Abstract

**Introduction:**

Inflammation is highly prevalent among patients with end-stage kidney disease and is associated with adverse outcomes. We aimed to investigate longitudinal changes in inflammatory markers in a diverse international incident hemodialysis patient population.

**Methods:**

The MONitoring Dialysis Outcomes (MONDO) Consortium encompasses hemodialysis databases from 31 countries in Europe, North America, South America, and Asia. The MONDO database was queried for inflammatory markers (total white blood cell count [WBC], neutrophil count, lymphocyte count, serum albumin, and C-reactive protein [CRP]) and hemoglobin levels in incident hemodialysis patients. Laboratory parameters were measured every month. Patients were stratified by survival time (≤6 months, >6 to 12 months, >12 to 18 months, >18 to 24 months, >24 to 30 months, >30 to 36 months, and >36 months) following dialysis initiation. We used cubic B-spline basis function to evaluate temporal changes in inflammatory parameters in relationship with patient survival.

**Results:**

We studied 18,726 incident hemodialysis patients. Their age at dialysis initiation was 71.3 ± 11.9 years; 10,802 (58%) were males. Within the first 6 months, 2068 (11%) patients died, and 12,295 patients (67%) survived >36 months (survivor cohort). Hemodialysis patients who died showed a distinct biphasic pattern of change in inflammatory markers where an initial decline of inflammation was followed by a rapid rise that was consistently evident approximately 6 months before death. This pattern was similar in all patients who died and was consistent across the survival time intervals. In contrast, in the survivor cohort, we observed initial decline of inflammation followed by sustained low levels of inflammatory biomarkers.

**Conclusion:**

Our international study of incident hemodialysis patients highlights a temporal relationship between serial measurements of inflammatory markers and patient survival. This finding may inform the development of prognostic models, such as the integration of dynamic changes in inflammatory markers for individual risk profiling and guiding preventive and therapeutic interventions.

## Graphical abstract

**Figure:**
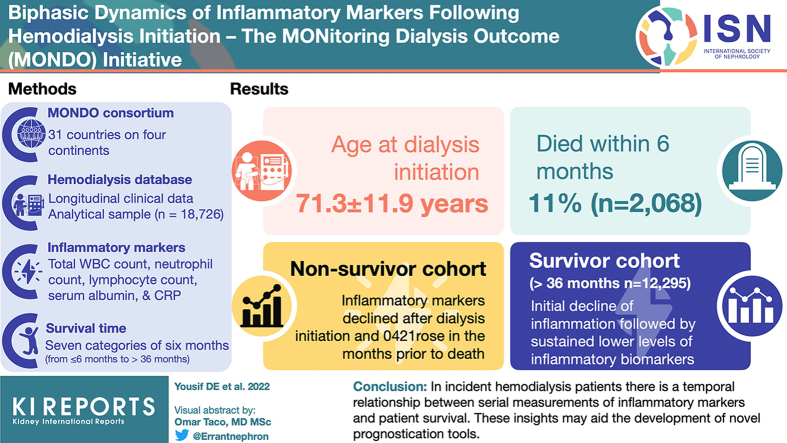

Mortality rates among chronic hemodialysis patients remain high despite technical and operational improvements over the past decades.^1^ The causes of excessive mortality among hemodialysis patients are multifactorial and have partly been attributed to heightened proinflammatory state and malnutrition.^2^, ^3^, ^4^ Inflammation in hemodialysis patients is due to multiple factors, including the uremic milieu, barrier breakdown (e.g., skin lesions, periodontitis, increased gut permeability), infections, decreased clearance of proinflammatory cytokines, fluid overload, endotoxemia, oxidative stress, high burden of prevalent cardiovascular disease, and other dialysis-related factors.^5^, ^6^, ^7^, ^8^

Over the past decade, several observational studies have demonstrated that increased inflammatory markers (elevated WBC, tumor necrosis factor-α, interleukin-6, and C-reactive protein [CRP]) and lymphopenia are associated with both cardiovascular and all-cause mortality among hemodialysis patients.^2^^,^^9^, ^10^, ^11^ Similarly, lower serum albumin levels are robustly associated with mortality in end-stage renal disease.^2^ We have recently reported that elevated neutrophil-to-lymphocyte ratio (NLR), a marker of systemic inflammation, is associated with mortality among incident hemodialysis patients.^12^ We have also shown that inflammatory markers, including higher NLR and lower albumin levels can be used as surrogate markers of systemic inflammation and are correlated with CRP.^13^ Nevertheless, most of these studies are cross-sectional in nature and there is scarcity of clinical studies evaluating longitudinal changes of inflammatory markers after dialysis initiation and their dynamic evolution before death.^14^, ^15^, ^16^ Understanding the temporal trends of inflammatory markers is important because it can inform us about unique pathophysiology and aid clinicians in identifying patients at greater risk for adverse outcomes where closer surveillance and earlier interventions might ultimately reduce mortality events.

Thus, in this study, we aimed to describe the dynamics of routinely measured inflammatory markers following hemodialysis initiation in a large and diverse international hemodialysis population and to explore their relationship with survival.

## Methods

### Study Population

Patients included in this study were part of the The MONitoring Dialysis Outcomes (MONDO) initiative. MONDO is a global, multicenter research collaboration that was designed to explore the determinants of patient survival in an international chronic kidney failure population, where longitudinal clinical data were collected in >130,000 patients from 31 countries on 4 continents (Asia, Europe, North and South America).^17^ The institutional review board at each of the participant institutions approved the study independently. Informed consent was obtained if required per local regulations. The participating consortia removed identifiable parameters before data transfer to the MONDO initiative in compliance with Health Insurance Portability and Accountability Act, the General Data Protection Regulation, and other locally applicable rules and regulations. The Western Institutional Review Board (ES-16-005) determined that this research activity in the MONDO database is exempt from institutional review board review.

This analysis was restricted to patients who started hemodialysis between January 1, 2000, and December 31, 2012 (study period). Only participants with recorded demographic information and contemporaneous monthly laboratory parameters (counts for WBC, neutrophils, and lymphocytes; serum albumin, CRP, and hemoglobin levels) were included in the analysis.

Total WBC, neutrophil and lymphocyte counts, and hemoglobin levels were determined using automated blood cell counters. Serum albumin was measured using the bromocresol green method. NLR was calculated as the ratio of neutrophil and lymphocyte counts. All measurements were done in local clinical laboratories as part of laboratory tests conducted on monthly basis for routine hemodialysis care. No in-patient laboratory data were included in the analysis.

### Statistical Analysis

Patients’ demographic information was obtained at dialysis initiation. Clinical and laboratory variables were averaged during the first 6 months on hemodialysis in patients who survived less than 6 months. Continuous variables were expressed as mean (SD) or median (interquartile range) and analyzed by parametric analysis of variance test or Kruskal-Wallis test, as appropriate. Categorical variables were expressed as relative frequency (%). We used cubic B-spline basis function curves to fit inflammatory markers (total WBC count, neutrophils, lymphocytes, NLR, CRP, and albumin) and hemoglobin levels from hemodialysis initiation until death or censoring event.

Patients were then stratified based on survival time in 7 categories (≤6 months, >6 to 12 months, >12 to 18 months, >18 to 24 months, >24 to 30 months, >30 to 36 months, and >36 months). Censoring events comprised lost to follow-up, transferred to clinics outside of MONDO partner networks, kidney transplantation, or recovery of renal function. Statistical analyses were conducted using SAS 9.4 (SAS Institute, Inc., Cary, NC) and R statistics software system, version 3.4.4 (R Foundation for Statistical Computing, Vienna, Austria). Plots of trajectories were created using ggplot2 (2.2.1.9000) packages (R Core Team, Hadley Wickham).

## Results

Of 36,078 MONDO participants, 17,352 participants with missing data on inflammatory markers were excluded. For final analysis, the study population comprised 18,726 incident hemodialysis patients with a mean (SD) age of 71.3 ± 11.9 years at hemodialysis start; 58% (*n* = 10,802) were men. The prevalence of diabetes was 61% (*n* = 11,435) (Table 1).

**Table 1:** Baseline characteristics of the study populations stratified by survival time

Survival time (mo)	≤6	(>6–12)	(>12–18)	(>18–24)	(>24–30)	(>30–36)	>36	*P* value
N	2068	1231	971	792	721	648	12,295	
Age (yr)	71.7 ± 11.7	70.7 ± 12.3	71.1 ± 12.1	70.6 ± 11.8	70.7 ± 12.2	70.2 ± 12.2	62.9 ± 14.3	<0.0001
Male (%)	58.0	60.0	59.0	58.0	59.0	58.0	58.0	0.43
Diabetes (%)	52.0	60.0	65.0	62.0	64.0	64.0	62.0	<0.0001
BMI (kg/m^2^)	25.8 ± 7.6	26.0 ± 9.4	27.2 ± 15.0	26.0 ± 6.3	26.6 ± 7.7	26.8 ± 9.9	26.8 ± 7.2	0.005
Treatment-related parameters								
IDWG (kg)	2.1 ± 2.1	2.2 ± 1.9	2.3 ± 2.0	2.2 ± 1.5	2.4 ± 2.1	2.4 ± 1.9	2.4 ± 2.0	<0.0001
Pre-HD SBP (mm Hg)	129.8 ± 22.0	133.9 ± 19.5	135.6 ± 19.9	136.5 ± 19.7	136.8 ± 18.9	137.2 ± 19.3	139.1 ± 18.3	<0.0001
eKT/V	1.2 ± 0.3	1.3 ± 0.3	1.3 ± 0.3	1.3 ± 0.3	1.3 ± 0.3	1.3 ± 0.3	1.3 ± 0.3	<0.0001
URR (%)	68.0 ± 10.8	70.5 ± 7.7	70.7 ± 7.5	71.2 ± 7.3	70.8 ± 7.3	70.5 ± 7.6	70.4 ± 7.3	<0.0001
nPCR (g/kg/d)	0.9 ± 0.2	1.0 ± 0.2	1.0 ± 0.2	1.0 ± 0.2	1.0 ± 0.2	1.0 ± 0.3	1.0 ± 0.3	<0.0001
Laboratory parameters								
WBC (10^9^/l)	8.8 ± 4.6	7.6 ± 3.1	7.5 ± 3.0	7.3 ± 3.4	7.3 ± 3.7	6.9 ± 2.8	7.1 ± 5.9	<0.0001
CRP (μg/ml)	32.5 ± 35.9	22.4 ± 26.9	18 ± 23.7	16.2 ± 21.4	14.9 ± 21.2	13.7 ± 20.1	10.2 ± 16.9	<0.0001
Neutrophils (%)	69.9 ± 13.4	65.6 ± 11.3	65.8 ± 9.7	64.4 ± 11.1	64.5 ± 10.6	63.8 ± 11.8	62.5 ± 11.5	<0.0001
Lymphocyte (%)	18.5 ± 10.4	21.6 ± 8.9	21.7 ± 7.8	21.7 ± 8.4	22.3 ± 8.7	21.5 ± 8.3	22.6 ± 8.8	<0.0001
NLR	6.9 ± 15.1	4.5 ± 4.4	4.1 ± 2.8	4.0 ± 3.2	4.3 ± 7.0	4.3 ± 5.5	3.8 ± 3.9	0.008
Hgb (g/dl)	9.4 ± 1.6	10.0 ± 1.5	10.2 ± 1.5	10.4 ± 1.5	10.5 ± 1.4	10.5 ± 1.4	10.7 ± 1.5	<0.0001
Serum albumin (g/dl)	3.2 ± 0.6	3.4 ± 0.5	3.5 ± 0.5	3.6 ± 0.5	3.6 ± 0.5	3.6 ± 0.5	3.7 ± 0.5	<0.0001
Serum creatinine (mg/dl)	5.9 ± 2.5	6.1 ± 2.2	6.1 ± 2.3	6.3 ± 2.2	6.2 ± 2.1	6.5 ± 2.3	7.2 ± 2.5	<0.0001
Serum phosphate (mg/dl)	4.2 ± 1.7	4.2 ± 1.5	4.2 ± 1.4	4.2 ± 1.5	4.2 ± 1.5	4.3 ± 1.5	4.4 ± 1.7	<0.0001
Total cholesterol (mg/dl)	154.1 ± 46.6	160.4 ± 47.1	161.3 ± 47.1	163.9 ± 45.4	161.2 ± 43.5	161.6 ± 44.2	169.7 ± 43.7	<0.0001

BMI, body mass index; CRP, C-reactive protein; eKT/V, equilibrated Kt/V; HD, hemodialysis; Hgb, hemoglobin; IDWG, intradialytic weight gain; nPCR, normalized protein catabolic rate; NLR, neutrophil-to-lymphocyte ratio; SBP, systolic blood pressure; URR, urea reduction ratio; WBC, white blood cell.

Baseline was defined as the first 6 months on dialysis.

There were notable differences in patient characteristics based on survival time (Table 1). Patients with shorter survival time were older and had higher levels of inflammatory markers at baseline as compared with the survivor cohort. Similarly, nonsurviving patients had lower body weight, blood pressure, and lower levels of hemoglobin, albumin, creatinine, phosphate and total cholesterol. In addition, equilibrated Kt/V and normalized protein catabolic rate were lower in patients with shorter survival time.

**Figure 1:**
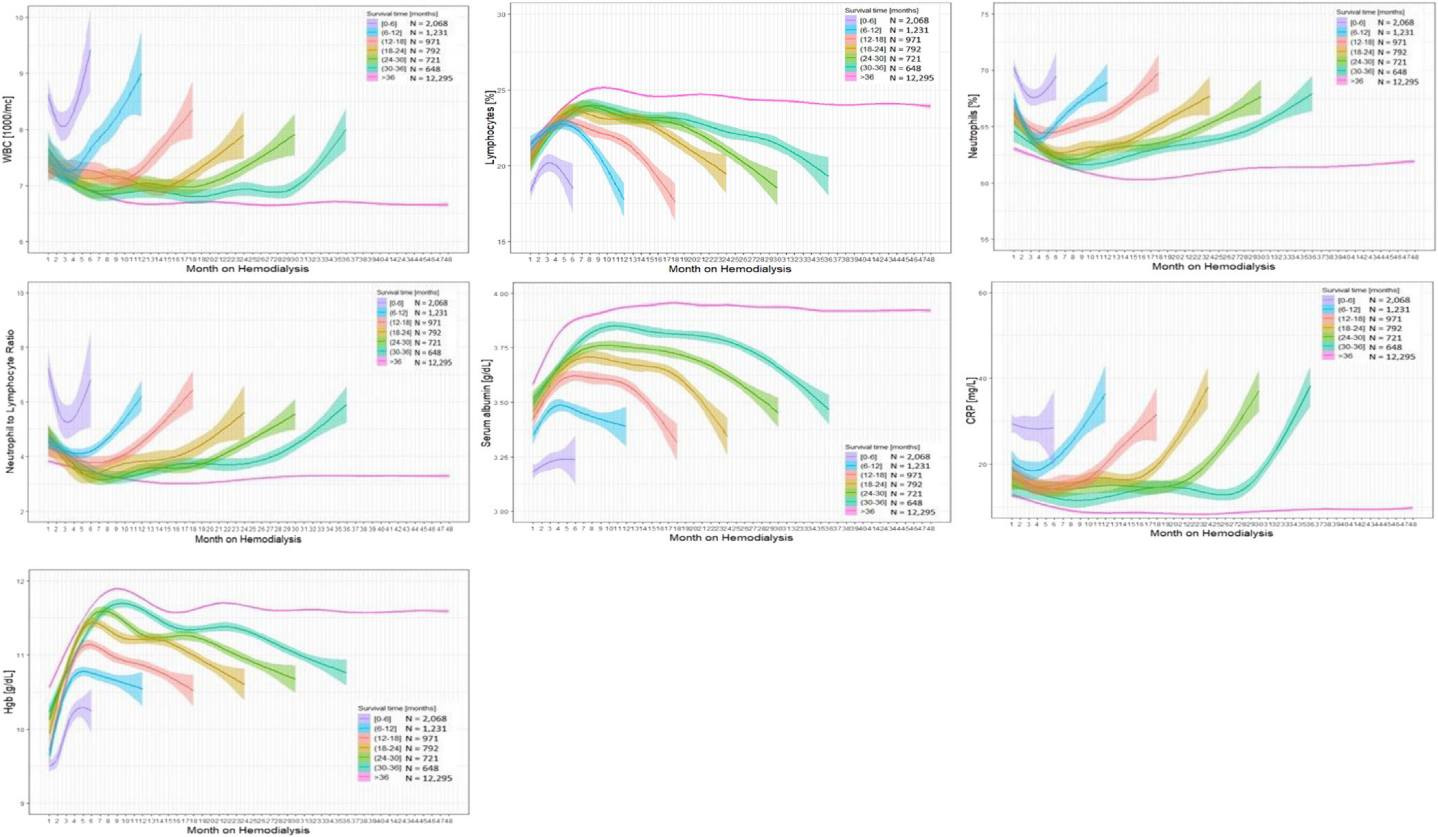
Trajectories of WBC, neutrophils, lymphocytes, NLR, serum albumin, CRP, and Hgb levels after hemodialysis initiation, stratified by survival time. The shaded area represents 95% confidence interval of the fit. CRP, C-reactive protein; Hgb, hemoglobin; WBC, white blood cell.

The temporal dynamics of inflammatory markers after hemodialysis initiation, stratified by survival time, is shown in Figure 1. Levels of inflammatory markers (WBC, neutrophils, NLR, CRP) decreased after dialysis initiation and increased in the months before death. When related to time to death, the inflection points appeared to occur consistently approximately 6 months before death. In contrast, lymphocyte count, serum albumin, and hemoglobin levels increased after dialysis initiation and decreased in the months preceding death. These patterns were qualitatively comparable independent of survival time in deceased patients. In contrast, those markers were notably different in hemodialysis patients who survived more than 36 months, where a decrease in levels of inflammatory parameters and an increase in serum albumin in the months after hemodialysis initiation were followed by a stable advancement over time. The pattern of inflammatory markers before survival was similar irrespective of age, race, and cause of death (data not shown).

## Discussion

Among a large sample of incident hemodialysis patients from 31 countries, we explored the dynamics of inflammatory markers and their relationship with mortality. Across markers, the inflammatory marker levels showed an early decrease after hemodialysis initiation that remained stable in survivors but consistently increased again before death in those who died. Interestingly, most inflammatory markers assessed here increased 6 months before death. Collectively, these findings hold important physiological and prognostic insights.

Chronic inflammation is prevalent in patients with kidney disease and may either reflect underling disease burden or contribute to higher morbidity and mortality among hemodialysis patients, or perhaps a combination of these 2 outcomes.^2^ Multiple studies have reported relationship of inflammatory markers with adverse outcomes in persons receiving maintenance hemodialysis.^3^^,^^9^, ^10^, ^11^, ^12^ To our knowledge, only a few prior studies have evaluated the relationship between dynamic changes of inflammatory markers with hospitalization and mortality in hemodialysis populations.^14^^,^^18^ One such study evaluated the dynamic patterns of laboratory and treatment parameters in a cohort of 52,180 hemodialysis patients (41,903 patients who died and 10,277 patients who survived for more than 4 years on hemodialysis).^14^ The investigators found that changes in serum albumin, intradialytic weight gain, systolic blood pressure, and CRP were observed 12 months before death.^14^ Our study corroborates the findings regarding CRP and albumin dynamics and extends the current knowledge because we evaluated additional inflammatory parameters before death. In addition, we also evaluated temporal trends of NLR from hemodialysis initiation in relation to survival time. NLR is a surrogate marker of systemic inflammation and previously shown to be more strongly associated with mortality as compared with total WBC count in hemodialysis patients.^12^ We found similar patterns of NLR change and other inflammatory markers. Finally, and most importantly, we stratified hemodialysis patients based on different survival periods after hemodialysis initiation, which allowed us to demonstrate that inflammatory markers behaved qualitatively similarly in patients with consistent inflections about 6 months before death, a pattern that was distinct in patients who survived for more than 36 months.

Inflammation also interacts with malnutrition, which collectively are part of a common protein-energy wasting syndrome.^6^ Some investigators believe that the malnutrition-inflammation complex is the strongest predictor of mortality and many other adverse health outcomes in hemodialysis patients, even stronger than the conventional risk factors.^19^^,^^20^ Serum albumin is influenced by inflammation, and numerous studies have shown a strong association between hypoalbuminemia and mortality.^19^^,^^20^ Thijssen *et al.*^21^ reported relatively rapid improvement in nutritional domains over the first few months on hemodialysis, followed by a gradual leveling off and eventually leading into a plateau toward the end of the second year. We also found improvement in serum albumin levels after start of hemodialysis and a decline in the months before death. Similar patterns of changes were observed for lymphocyte count and hemoglobin levels. The improvements in serum albumin and hemoglobin levels after hemodialysis initiation is attributed to decreased inflammation, improved nutrition, and fluid status.

Our study has clinical and research implications. Understanding the temporal patterns of the inflammatory milieu changes can provide important insight into pathophysiological mechanisms and dysregulation events before death in highly vulnerable hemodialysis populations. The incorporation of dynamic changes of readily available inflammatory markers in the electronic databases can allow early and timely detection of hemodialysis patients who are at high risk of death. Whereas the changes observed here may be relatively subtle and perhaps easy to miss in individual patients over time, the evaluation of multiple biomarkers of inflammation and higher-level data structures is now feasible with artificial intelligence and other similar methods. The systematic availability of these laboratory data may allow novel statistical approaches to identify individual patients at higher risk of adverse outcomes, allowing early alerting to dialysis physicians and staff. For example, such alerts may identify individuals with occult infections or early malnutrition. Furthermore, alert systems can be developed to support surveillance, early management, and patient counseling, and whether such interventions may ultimately translate to improved clinical outcomes.

Our study has several strengths. Its large patient population, all among incident hemodialysis patients, the well-characterized and fairly heterogeneous cohort across multiple countries enhance the generalizability of our research findings. Stratification by time to death rather than from dialysis initiation elucidated consistent patterns of biomarker changes approximately 6 months before death that may have been missed using standard time-to-event analysis from study enrollment. The availability of multiple biomarkers of inflammation highlighted the consistency of findings, gives important insight into the underlying biology, and makes it clear that the associations are not related to any one biomarker, but rather a systemic process linked with comprehensive changes across the biomarkers evaluated. Our study also has some limitations. First, it is observational and retrospective in nature. The study was also restricted to incident hemodialysis participants who have complete available data of inflammatory markers.

In summary, our study revealed biphasic dynamic changes in inflammatory markers with a decrease after hemodialysis initiation and an increase in the months before death. These insights may aid the development of novel prognostication tools, which may aid clinicians in identifying hemodialysis patients at the highest risk of death in the short term to medium term, where closer surveillance may be warranted.

## Disclosure

SS, JB, AMG, and LAU are employees of Fresenius Medical Care Global Medical Office. PK, XY, and JR are employees of Renal Research Institute, a wholly own subsidiary of Fresenius Medical Care North America. JPK and FMvdS are employees of Maastricht University Medical Center and have received research grants from Fresenius Medical Care. PK holds stock in Fresenius Medical Care NA and receives author royalties from HSTalks. JI has served as an expert consultant from Akebia, AstraZeneca, Bayer, Jnana, AlphaYoung, and Ardelyx biopharmaceuticals; he is the principal investigator of an investigator-initiated research grant from Baxter International and serves as a Data and Safety Monitoring Board member for a trial supported by Sanifit International. CP work at Imperial College was supported by the National Institute for Health and Care Research Imperial Biomedical Research Center.

## References

[1] de Jager D.J., Grootendorst D.C., Jager K.J. (2009). Cardiovascular and non-cardiovascular mortality among patients starting dialysis. JAMA.

[2] Filiopoulos V., Hadjiyannakos D., Takouli L. (2009). Inflammation and oxidative stress in end-stage renal disease patients treated with hemodialysis or peritoneal dialysis. Int J Artif Organs.

[3] Zimmermann J., Herrlinger S., Pruy A. (1999). Inflammation enhances cardiovascular risk and mortality in hemodialysis patients. Kidney Int.

[4] Foley R.N., Parfrey P.S., Harnett J.D. (1996). Hypoalbuminemia, cardiac morbidity, and mortality in end-stage renal disease. J Am Soc Nephrol.

[5] Dekker M.J., Marcelli D., Canaud B.J. (2017). Impact of fluid status and inflammation and their interaction on survival: a study in an international hemodialysis patient cohort. Kidney Int.

[6] Aram M.M., Fein P.A., Rafiq M.A., Schloth T., Chattopadhyay J., Mittman N. (2006). Malnutrition and inflammation as predictors of mortality in peritoneal dialysis patients. Kidney Int.

[7] Rocco M.V., Dwyer J.T., Larive B. (2004). The effect of dialysis dose and membrane flux on nutritional parameters in hemodialysis patients: results of the HEMO Study. Kidney Int.

[8] Lopez-Gomez J.M., Villaverde M., Jofre R. (2005). Interdialytic weight gain as a marker of blood pressure, nutrition, and survival in hemodialysis patients. Kidney Int Suppl.

[9] Yeun J.Y., Levine R.A., Mantadilok V., Kaysen G.A. (2000). C-reactive protein predicts all-cause and cardiovascular mortality in hemodialysis patients. Am J Kidney Dis.

[10] Kuwae N., Kopple J.D., Kalantar-Zadeh K. (2005). A low lymphocyte percentage is a predictor of mortality and hospitalization in hemodialysis patients. Clin Nephrol.

[11] Lichtenberg S., Korzets A., Zingerman B. (2015). An intradialytic increase in serum interleu-kin-6 levels is associated with an increased mortality in hemodialysis patients. Int J Artif Organs.

[12] Ouellet G., Malhotra R., Penne E.L. (2016). Neutrophil-lymphocyte ratio as a novel predictor of survival in chronic hemodialysis patients. Clin Nephrol.

[13] Malhotra R., Marcelli D., von Gersdorff G. (2015). Relationship of neutrophil-to-lymphocyte ratio and serum albumin levels with C-reactive protein in hemodialysis patients: results from 2 international cohort studies. Nephron.

[14] Usvyat L.A., Barth C., Bayh I. (2013). Interdialytic weight gain, systolic blood pressure, serum albumin, and C-reactive protein levels change in chronic dialysis patients prior to death. Kidney Int.

[15] Ye X., Dekker M.J., Maddux F.W. (2017). Dynamics of nutritional competence in the last year before death in a large cohort of US hemodialysis patients. J Ren Nutr.

[16] Kotanko P., Thijssen S., Usvyat L. (2009). Temporal evolution of clinical parameters before death in dialysis patients: a new concept. Blood Purif.

[17] Usvyat L.A., Haviv Y.S., Etter M. (2013). The monitoring dialysis outcomes (MONDO) initiative. Blood Purif.

[18] Usvyat L.A., Kooman J.P., van der Sande F.M. (2014). Dynamics of hospitalizations in hemodialysis patients: results from a large US provider. Nephrol Dial Transplant.

[19] Danielski M., Ikizler T.A., McMonagle E. (2003). Linkage of hypoalbuminemia, inflammation, and oxidative stress in patients receiving maintenance hemodialysis therapy. Am J Kidney Dis.

[20] Bradbury B.D., Fissell R.B., Albert J.M. (2007). Predictors of early mortality among incident United States hemodialysis patients in the dialysis outcomes and practice patterns study (DOPPS). Clin J Am Soc Nephrol.

[21] Thijssen S., Wong M.Y., Usvyat L.A. (2015). Nutritional competence and resilience among hemodialysis patients in the setting of dialysis initiation and hospitalization. Clin J Am Soc Nephrol.

